# Researching progress on bio-reactive electrogenic materials with electrophysiological activity for enhanced bone regeneration

**DOI:** 10.3389/fbioe.2022.921284

**Published:** 2022-07-25

**Authors:** Shaojie Dong, Yuwei Zhang, Yukun Mei, Yifei Zhang, Yaqi Hao, Beilei Liang, Weijiang Dong, Rui Zou, Lin Niu

**Affiliations:** ^1^ Key Laboratory of Shaanxi Province for Craniofacial Precision Medicine Research, College of Stomatology, Xi’an Jiaotong University, Xi’an, China; ^2^ Clinical Research Center of Shaanxi Province for Dental and Maxillofacial Diseases, Xi’an, China; ^3^ Department of Prosthodontics, College of Stomatology, Xi’an Jiaotong University, Xi’an, China; ^4^ School of Basic Sciences of Xi’an Jiaotong University Health Science Center, Xi’an, China

**Keywords:** electrogenesis, bone regeneration, electrical stimulation, bio-reactive material, electrophysiological activity

## Abstract

Bone tissues are dynamically reconstructed during the entire life cycle phase, which is an exquisitely regulated process controlled by intracellular and intercellular signals transmitted through physicochemical and biochemical stimulation. Recently, the role of electrical activity in promoting bone regeneration has attracted great attention, making the design, fabrication, and selection of bioelectric bio-reactive materials a focus. Under specific conditions, piezoelectric, photoelectric, magnetoelectric, acoustoelectric, and thermoelectric materials can generate bioelectric signals similar to those of natural tissues and stimulate osteogenesis-related signaling pathways to enhance the regeneration of bone defects, which can be used for designing novel smart biological materials for engineering tissue regeneration. However, literature summarizing studies relevant to bioelectric materials for bone regeneration is rare to our knowledge. Consequently, this review is mainly focused on the biological mechanism of electrical stimulation in the regeneration of bone defects, the current state and future prospects of piezoelectric materials, and other bioelectric active materials suitable for bone tissue engineering in recent studies, aiming to provide a theoretical basis for novel clinical treatment strategies for bone defects.

## 1 Introduction

Critical-sized bone defects caused by cleft palate, trauma, infection, tumors, and other genetic or environmental factors exhibit a non-negligible incidence rate and remain difficult to regenerate (Geng et al., 2021; Westhauser et al., 2021; Li et al., 2022). Traction osteogenesis, autologous bone transplantation, and allogeneic bone transplantation are commonly used for bone restoration in the clinic, whereas the effect of these methods is restricted by insufficient sources, bone lesions, secondary injury, potential antigenicity, infection, and other undefined concerns of donors (Terpos et al., 2021).

With the prosperous development of biomaterials, new possibilities for the restoration of massive bone defects have been provided. Among them, bone-implanted biomaterials can be roughly sorted into three generations, referred to as bioinert materials, bioactive materials, and bio-reactive materials. As the first generation of biomaterials, bioinert materials were initially developed during the 1960s–1980s, which included artificial joints, bone nails, and bone plates for internal fixation. These inert biomaterials, which are most widely used in humans, exhibit attractive biocompatibility in the long term during the post-implanted stage (Senra and Marques, 2020). Since traditional inanimate medical metals, polymers, ceramics, and other conventional materials make no adaptive response to changes in bone defects, they can hardly meet the actual needs in the clinic or fulfill the requirements of developing modern biomaterials. Thus, researchers in the field of biomedical materials had started to shift their attention from biocompatibility to bioactivity in the 1980s. Therefore, the second generation of biomedical materials was proposed, which were characterized by controllable degradation, ion exchange capability, polycondensation, and even stimulation of osteoblasts under physiological conditions, ultimately contributing to the formation of new bone (Im, 2020). However, the aforementioned biomaterials have no bidirectional interaction with bone tissues under physiological load or biochemical stimulation, which is a common disadvantage. In recent years, the requirement for new strategies for treating bone defects has increased, leaving new opportunities and challenges for the development of next-generation biomedical materials. Significantly, to keep with the progress of biology and biomaterial science, researchers are forging ahead to incorporate this new opportunity into specific groundbreaking biomaterials. Discoveries in molecular biology are now opening new frontiers for the design of biomaterials and brand paths to biomaterials that will work with normal physiology and integrate into the human body seamlessly (Gocha and Mcdonald, 2020; Kim et al., 2021). In addition to the innate functions of supporting, replacing, and restoring, new properties, such as biologically inducing activity, would be incorporated. In this context, third-generation bio-reactive materials that can transfer environmental factors to specific signals to induce cell responses at the molecular level have been proposed. These bio-reactive materials can trigger reactions with osteogenesis-related cell integrin proteins and induce proliferation, differentiation, and secretion of extracellular matrix (ECM) to promote tissue regeneration. Therefore, simulating body tissue composition and structure to realize functional simulation has become the mainstream direction of third-generation biomaterials (Gaharwar et al., 2020).

Nevertheless, third-generation materials still have the potential for further improvement. With the gradual clarification of the physiological mechanism of bone restoration and electrical response, bioelectric active materials have been increasingly studied and have shown fascinating effects and potential. Relevant studies have revealed the existence of both piezoelectricity and inverse piezoelectricity in the bone, which may be key factors in the bone healing process enhanced by low-intensity biophysical–electric stimulation. Meanwhile, with the recognition of wound electrical activity as a long-lasting and regulated response, it was demonstrated that natural, endogenous electric fields and electric current could arise spontaneously after the wound of tissues, which might be necessary for the healing of defects (Cheah et al., 2021; Wang et al., 2022). Regretfully, it is rarely found that the design, classification, and application of existing or potential bioelectric materials that can be utilized for the restoration of bone defects are systemically researched. Therefore, the functional mechanisms, synthetic pathways, and application of electrogenic materials in the field of bone regeneration are reviewed herein, aiming to provide a comprehensive perspective for the design, fabrication, and utilization of novel bioelectric active materials.

## 2 Electrical stimulation enhanced bone regeneration

The electroactivity of biological tissue is the basic attribute of life phenomena and plays an irreplaceable role in the process of metabolism (Chiranjeevi and Patil, 2020). There is also much evidence of endogenous electrical signals that play key roles in regulating the development and regeneration of many tissues (So et al., 2020; Casella et al., 2021). With the further clarification of bone tissue composition, it has been found that natural bone tissue is closely related to bioelectricity, that is, the bone has the same electroactivity as other living tissues. Half a century ago, the piezoelectric property of the bone was first reported (Fukada and Yasuda, 1964). Since the application of electrical stimulation in the treatment of tibial anterior foot ptosis in the 1960s (Liberson et al., 1961), after years of technical innovation, electrical stimulation technology has become a reliable method to treat patients with paralysis.

Electrical stimulation, such as pulsed electromagnetic field, pulsed alternating current, direct current, and electrostatic field, has been proven to be beneficial to the growth and repair of new bone in many animal experiments and clinical practices (Khatua et al., 2020; Sahm et al., 2020; Devet et al., 2021). Clinically, electrical stimulation osteogenesis can be roughly divided into implantation and non-implantation methods. In the first method, all or a part of the electrical stimulation device is implanted into a wound through surgery, which belongs to invasive electrical stimulation. After treatment, the electrode often needs to be removed, which usually leads to infection and secondary injury; in the second method, it is generally the external electrical stimulation of the fracture part and belongs to noninvasive electrical stimulation. It can neither stimulate the fracture site accurately nor guaranty the effectiveness of stimulation.

Subsequently, it has been proven that bioelectrical signals, endogenous electric field, and external electrical stimulation play an important role in regulating cell behavior in the field of bone repair, such as osteoblasts, chondrocytes, fibroblasts, and smooth muscle cells (Yu et al., 2021; Wei et al., 2022). Therefore, researchers are looking for electroactive materials that can simulate the microenvironment and transmit signals that stimulate osteoblast-related cell responses.

In recent years, numerous innovative biomaterials that can accelerate regenerating effects by transmitting physiological electrical cues and enhancing the electrical environment without external stimulation devices have been found. Thus, an opportunity for the local cure of diseases is provided by accurately regulating cell behavior. Optical, electrical, ultrasonic, and magnetic external energy have great potential to trigger electronic stimulations due to their noninvasive and accurate characteristics (Kang et al., 2018; Zhang et al., 2019; Trinh et al., 2020).

## 3 Current and potential electrogenic materials for enhanced bone regeneration

### 3.1 Piezoelectric materials

The phenomenon of “piezoelectricity,” which means pressure—originating from the Greek word “piezein,” was first discovered in 1880 (Mould, 2007). With the proposal of “Wolf’s law,” people began to realize that complexes composed of collagen fibrils with a dense arrangement of hydroxyapatite particles in bone tissue would reshape their structure in response to external stress. Moreover, the latest studies suggest that various biological tissues, such as keratin, tendons, cartilage, dentin, and cementum, possess remarkable piezoelectric properties, which may be attributed mainly to the components of collagen (Kim et al., 2020). Due to the piezoelectric property, collagen can generate electrical signals in response to loaded forces. For instance, dense bone contains a large amount of type I collagen, and its piezoelectric constant is approximately 0.7 pC/N (Puppi et al., 2010).

Piezoelectric materials, as their name implies, are characterized by the piezoelectric effect, which can convert mechanical pressure into an electrical signal (Zaszczynska et al., 2020). As a sensitive mechanical–electrical transduction platform, piezoelectric materials can utilize physiological deformation of bone tissue with movement to generate instant bioelectric stimulation and transfer it into biomimetic electrophysiological signals to obtain appropriate physiological functions (Kapat et al., 2020).

**FIGURE 1:**
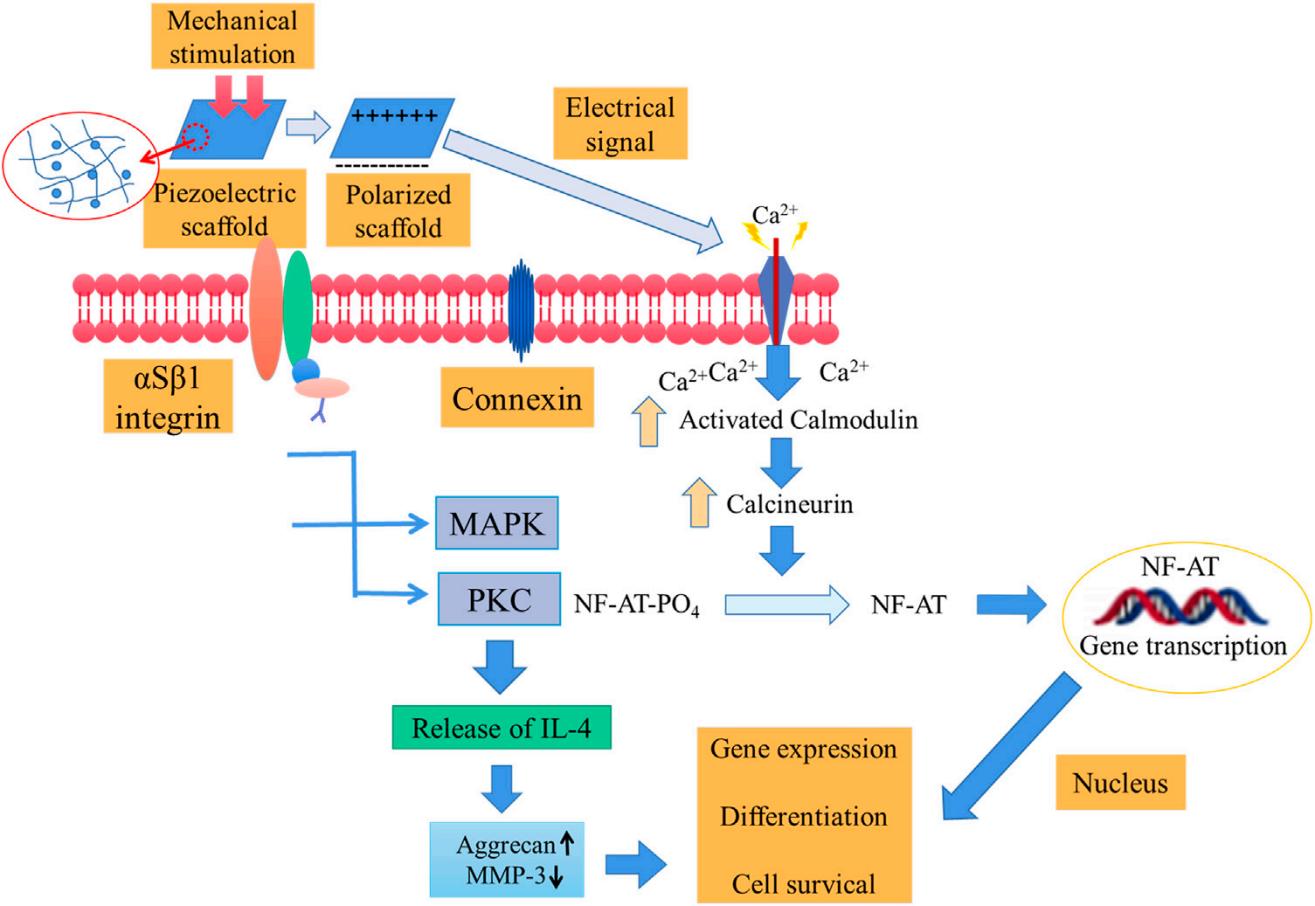
Schematic diagram of the activation of signal transduction pathways in response to electrical and mechanical stimulation. Mechanical stimulation is transformed into electrical signals to activate voltage-gated Ca^2+^ channels. The further increase in the intracellular Ca^2+^ concentration activates the calcium-modulated protein which then further activates calcineurin (calcium and calmodulin-dependent serine/threonine protein phosphatase). The activated calcineurin can dephosphorylate NF-AT and transfer it to the nucleus, where it acts as a transcription factor together with other related proteins. Additionally, mechanical stimulation can activate mechanical receptors in the membrane, thereby activating PKC and MAPK signaling cascades. These cascades lead to the synthesis of proteoglycans, and the inhibition of IL-1 and proteoglycans can be broken down (Jacob et al., 2018).

The *in vivo* working mechanism of piezoelectric materials determines their clinical value to a large degree. Electroactive piezoelectric scaffolds simulating the piezoelectric coefficient of natural tissue can generate electrical signals along with the loaded stress, and the generated charge and electric dipole stimulates bone remodeling and growth by opening the voltage-gated calcium channel (Figure 1). The calcium/calmodulin pathway of osteocytes thus activated to produce transforming growth factor β and act on osteoblasts, osteoclasts, and other cells promotes osteogenic differentiation, proliferation, and tissue restoration (Jacob et al., 2018).

**FIGURE 2:**
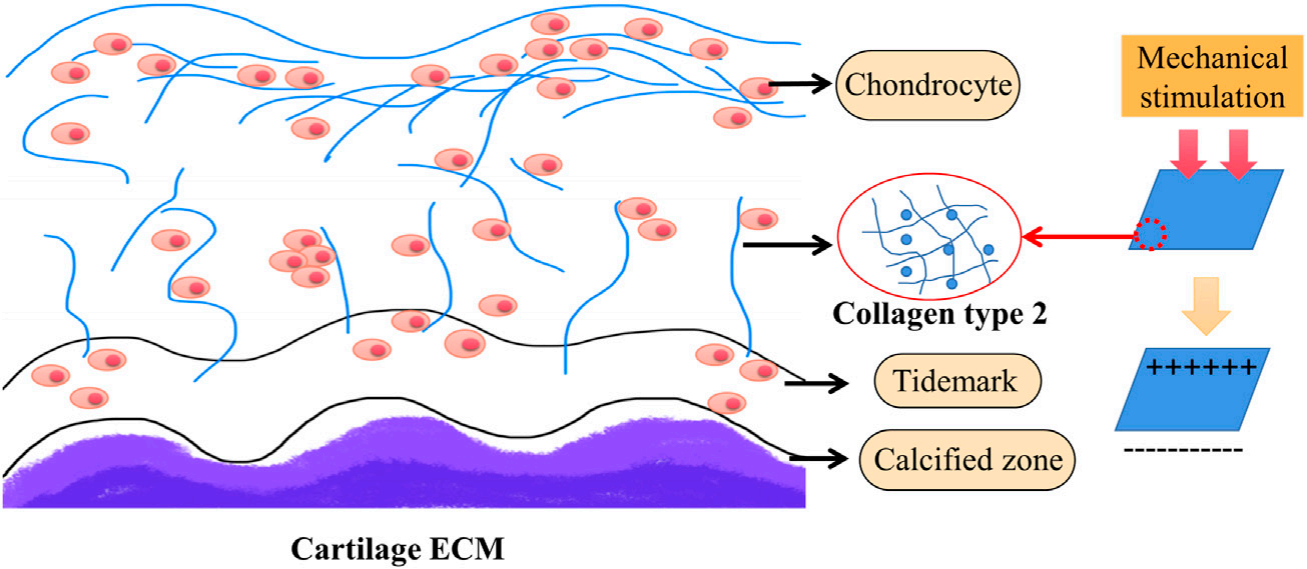
The mechanism of cartilage regeneration. Cartilage is rich in type II collagen. Piezoelectric collagen affects cell membrane receptors with changes in charge and ultimately acts on the nucleus to promote cartilage regeneration (Jacob et al., 2018; Jacob et al., 2019).

Since piezoelectric type II collagen is abundant in cartilage, it affects cell membrane receptors with changes in electric charge and finally acts on the nucleus to promote cartilage regeneration (Figure 2) (Reddi and, 2000; Jacob et al., 2019; Zhang et al., 2020).

**FIGURE 3:**
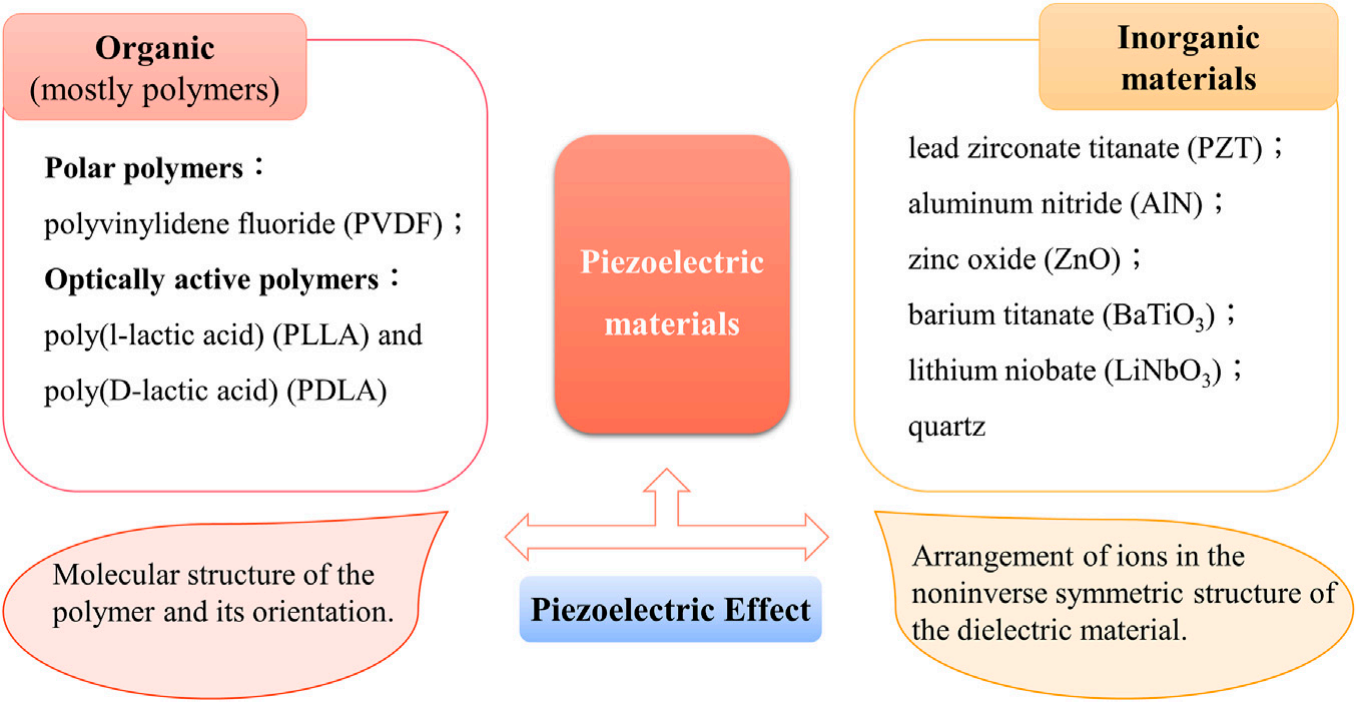
Representative materials of the piezoelectric material family and their causes of the piezoelectric effect. Piezoelectric materials are a family of organic and inorganic materials (Chorsi et al., 2019).

Piezoelectric materials are most concerned about the polarization ability of piezoelectric bodies under pressure. Therefore, it is necessary to introduce the most commonly used piezoelectric constant (“d_ij_” constant) which reflects the linear response relationship between the piezoelectric physical quantity and electrical quantity. The first subscript “i” represents the direction of polarization (or applied electric field) and the second subscript “j” represents the direction of applied stress (or induced strain). It is the expression of the amount of charge generated by the material on the applied stress and the strain experienced by the material applying the unit electric field. Piezoelectric materials are a family of both organic (mostly polymers) and inorganic materials that can convert mechanical force into electricity and *vice versa* (Figure 3). Piezoelectric materials can be roughly divided into piezoelectric polymers and piezoelectric ceramics, as mentioned above, which can be used alone or together in tissue engineering. Concretely, it is also classified into four different categories: 1) naturally occurring piezocrystals, 2) piezoceramics (titanates, lead-based, and lead-free ceramics), 3) piezopolymers, and 4) piezocomposites (Kapat et al., 2020). Next, we will take piezoelectric polymer and piezoelectric ceramics as the main representatives to introduce piezoelectric biomaterials.

#### 3.1.1 Piezoelectric polymer

Piezoelectric polymers include PVDF (polyvinylidene fluoride), PHBV (poly-3-hydroxybutyrate-3-hydroxyvalerate), polyamides, poly-l-lactic acid (PLLA), and biopolymers such as cellulose, collagen, chitin, and others. Piezoelectric polymers have the advantage of processing flexibility, which represents higher strength and impact resistance than inorganic materials (Jacob et al., 2018). To obtain the characteristics of piezoelectricity, piezoelectric polymers often require elaborately designed permanent molecular dipoles, the ability to align or orient molecular dipoles, maintain alignment once achieved, and experience large strains under mechanical stress (Fousek et al., 1999; Xu et al., 2018).

PVDF is one of the most famous piezoelectric copolymers with a piezoelectric coefficient of 20 pC/N and five crystal polymorphisms, namely, the nonpolar α- (most common), β-, γ-, δ-, and ε-phases (Li et al., 2019a). The β-PVDF phase shows excellent piezoelectric and ferroelectric properties. Because of its high flexibility and nontoxicity, PVDF has been used in various ways, from tissue engineering scaffolds to implantable automatic force devices and other biomedical applications (Hamzah et al., 2021). After corona polarization of PVDF scaffolds, the negatively charged surface that is formed promotes better adhesion and proliferation of myoblasts (Martins et al., 2013). When mechanically stimulated, the surface of piezoelectric PVDF generates an electric current, which enhances the proliferation and osteogenic differentiation of osteoblasts and adipose stem cells (Zhang et al., 2018). The *in vivo* evaluation results of the piezoelectric PVDF film confirmed its applicability as a candidate for bone-repairing substitutes (Wang et al., 2018).

Problems of improving hydrophilicity are an obstacle for PVDF in serving as a biological piezoelectric coating in bone defect repair. Thus, a polarized HA/PVDF coating is prepared to reduce the contact angle of the material by 66.9%. When the piezoelectric coefficient of the 20-pC/N HA/PVDF biological piezoelectric coating reaches 1.52 pC/N, the contact angle of the coating becomes 31.7° in the process of contact with the body fluid or tissue fluid, which is lower than the surface of the previous hydrophobic material and would greatly reduce the adsorption of cells and proteins on the surface that is conducive to tissue restoration at the later stage (Wu et al., 2020). In addition, when the charge is not high enough to cause a beneficial cellular response, it is necessary to improve the piezoelectric properties of the scaffold. Researchers manufactured core-shell composite submicron fibers of PVDF with the addition of graphene oxide and reported that the piezoelectric constant increased by 426% when compared with neat PVDF fibers (Diabor et al., 2019). It was found that when the content of graphene oxide was higher than 0.1 wt.%, the composition of PVDF in the (GO)/PVDF nanocomposite films would have a phase transition from the mixed state of the α- and β- to the high purity state of the β-phase. When the content of graphene oxide was higher than 2 wt.%, the Young’s modulus and tensile strength of PVDF increased by 192 and 92%, respectively (El Achaby et al., 2012).

P(VDF-TrFE) are copolymers of vinylidene fluoride (VDF) and trifluoroethylene (TrFE), possessing ideal cell compatibility and conduciveness to trigger the integrin-mediated FAK signaling pathway, finally upregulating the osteogenic differentiation of MSCs (Shen et al., 2021; Zhu et al., 2022). The piezoelectric coefficient of the copolymers reached the highest value of 30 pC/N (Fukada, 1998). PVDF and PVDF TrFE have been mixed with starch or cellulose, similar to natural polymers, to produce porous structures conducive to tissue growth to develop scaffold structures suitable for tissue restoration and regeneration, especially for bone tissue engineering (Pereira et al., 2014). Polyamides have limited application in tissue engineering due to difficult degradation, which needs to be further modified. Modified polyamide–hydroxyapatite composite can promote osteogenesis after 12 weeks of implantation (Wang et al., 2007).

Natural biopolymer polymers are becoming increasingly important in tissue engineering because of their degradability and low toxicity, which are conducive to biological signal transduction, cell adhesion, and cell response. However, their physical properties are not sufficient, and their biological properties may be lost in the process of mixing with other materials. In addition, appropriate screening and treatment are needed to avoid disease transmission and immune rejection. Appropriate chemical or physical treatment helps overcome the above problems. Cellulose is the most abundant natural polymer on Earth, with excellent biocompatibility, high tensile strength, and shear properties (Diabor et al., 2019; Dou et al., 2021). The shear piezoelectric coefficient (d_14_), which is a representative indicator of high tensile strength, is 0.2 pC/N (Kim et al., 2006). Although small pore size or dense reticular fibers limit cell infiltration, it can be improved by adding an appropriate pore-forming agent. In addition, studies have shown that cellulose has the ability to promote cell adhesion, especially chondrocytes, osteocytes, endothelial cells, and smooth muscle cells (Zaborowska Bodin et al., 2010). Therefore, it is a piezoelectric material suitable for bone and cartilage tissue engineering. Collagen is a biological protein and an important part of the bone, cartilage, tendons, teeth, and blood vessels. It has the desired biocompatibility, cell binding performance, hydrophilicity, low antigenicity, and *in vivo* absorption capability and has been used in the field of bone and cartilage regeneration. However, the limitations of collagen, such as low mechanical stiffness, rapid degradation, and toxicity of adding a crosslinking agent, should be overcome before it can be widely applied in the clinic (Jacob et al., 2018).

#### 3.1.2 Piezoelectric ceramics

Piezoelectric ceramics are polycrystalline (Narayan et al., 2018) and have a high piezoelectric coefficient, for example, barium titanate, zinc oxide, potassium sodium niobate (KNN), lithium sodium potassium niobate (LKNN), and boron nitride. Generally, inorganic piezoelectric materials are biocompatible or can be biocompatible after being encapsulated, while lead-based ceramics have limited applications in tissue engineering due to their cytotoxicity. Lead-free piezoelectric ceramics may be another option. Other ceramics also have dose-dependent toxicity, which are suitable for tissue engineering to a certain extent. Lead-free piezoelectric ceramics can be divided into five systems, such as the barium titanate, niobate base, bismuth layer, sodium bismuth titanate, and tungsten bronze systems. Among them, the barium titanate system and the niobate system are the most widely studied. The former is well known in relevant theories and applications, and the latter niobate ceramics have been developed since 2004, when the modification of lithium, tantalum, and antimony in the KNN ceramic texture structure experienced breakthroughs and attracted much attention (Saito et al., 2004). Barium titanate (BTO) is a piezoelectric ceramic with high biocompatibility that was independently discovered by American scientists and former Soviet scientists in 1946, the d_33_ coefficient of which reached 191 pC/N (Mindlin, 1972). The piezoelectric property of BTO was first discovered as a sort of strong dielectric compound material with a high dielectric constant and low dielectric loss. Due to its early discovery, stable chemical properties and good piezoelectric properties, BTO occupied the leading position in early piezoelectric materials and became a research hotspot. Yamashita et al. (1996) soaked polarized barium titanate and HA composite (HABT) ceramics in simulated body fluid (SBF) for different times. The results show that a large number of bone-like apatite crystals were formed on the negatively charged surface, while only NaCl was deposited on the surface of the opposite electrode. Hwang et al. (2002) further studied the osteoapatite-inducing ability of barium titanate ceramics with different polarization degrees. Similar results were obtained when polarized barium titanate was placed in simulated body fluid (SBF). It was found that the higher the degree of polarization was, the greater the calcium phosphorus ratio would be, which could be attributed to the electrostatic adsorption effect (Masataka et al., 2001).

As an electroactive biomaterial, barium titanate ceramics have poor temperature stability and are liable to deteriorate, preventing the cytotoxicity of titanium and barium ions from being ignored under long-term physiological conditions. These shortcomings limit its further application in the biological field.

Lead-free niobate piezoelectric materials, as previous studies have indicated, enhance the proliferation and osteogenic activity of osteoblasts for rapid bone regeneration. To ensure adequate mechanical strength and piezoelectric properties of the piezoelectric ceramics, Li is added. The relatively considerable biocompatibility of ferroelectric lithium niobate (LN) has been demonstrated by culturing and fluorescence imaging MC3T3 osteoblast cells for up to 11 days. While rapid bone regeneration was discovered, mineralization was observed for all LN surfaces at 20 days, whereas no mineral was observed on electrostatically neutral control glass surfaces until day 30. Qualitatively, for 30 and 40 days, there appears to be more mineralization on charged than on uncharged surfaces (Carville et al., 2015). KNN and LKNN are also lead-free Li-doped piezoelectric ceramics with piezoelectric coefficients of 63 pC/N and 98 pC/N, respectively. When exposed to the bioenvironment, the cytotoxicity of Li would slightly increase during the release process when compared with that of KNN (Yu et al., 2012). After all, the addition of Li could be a mixed blessing, under the condition of slight loss of biocompatibility, Li improves the strength and piezoelectric properties of the material, which is worthy of medical implantation and long-term electrophysiological osteogenesis.

### 3.2 Optoelectronic materials

As a highly orthogonal external stimulus, light has the unique ability to accurately manipulate the cellular signal system, which has been widely used in materials science, chemistry, biology, and drug delivery systems. In particular, light can be used as a noninvasive external energy source to monitor and trigger the spatiotemporal dynamics of cell signals. To regulate the cell signal transduction process, attempts have been made to covalently connect photostability chemical groups to signal molecules necessary for cell function (Zhu et al., 2018; Tang and Wang, 2021; Wakiyama et al., 2021). In search of suitable and stable donors, optoelectronic materials have come into sight during this exploring process.

Nevertheless, not all light can penetrate through deep tissue or be harmless to healthy histocytes. Only a few types of electromagnetic waves with specific wavelengths have harmless tissue penetration properties; among them, near infrared (NIR, wavelength from 780 to 2,526 nm) light irradiation has been demonstrated to cause negligible damage to cells when compared to other short wavelength lasers that can produce phototoxic side effects. Moreover, NIR light is absorbed mainly by nanoparticles that react to NIR light and barely by water or surrounding tissues. In addition, this whole process is difficult to interfere with by solvents or the surrounding environment (Zhang et al., 2016; Li et al., 2019b).

**FIGURE 4:**
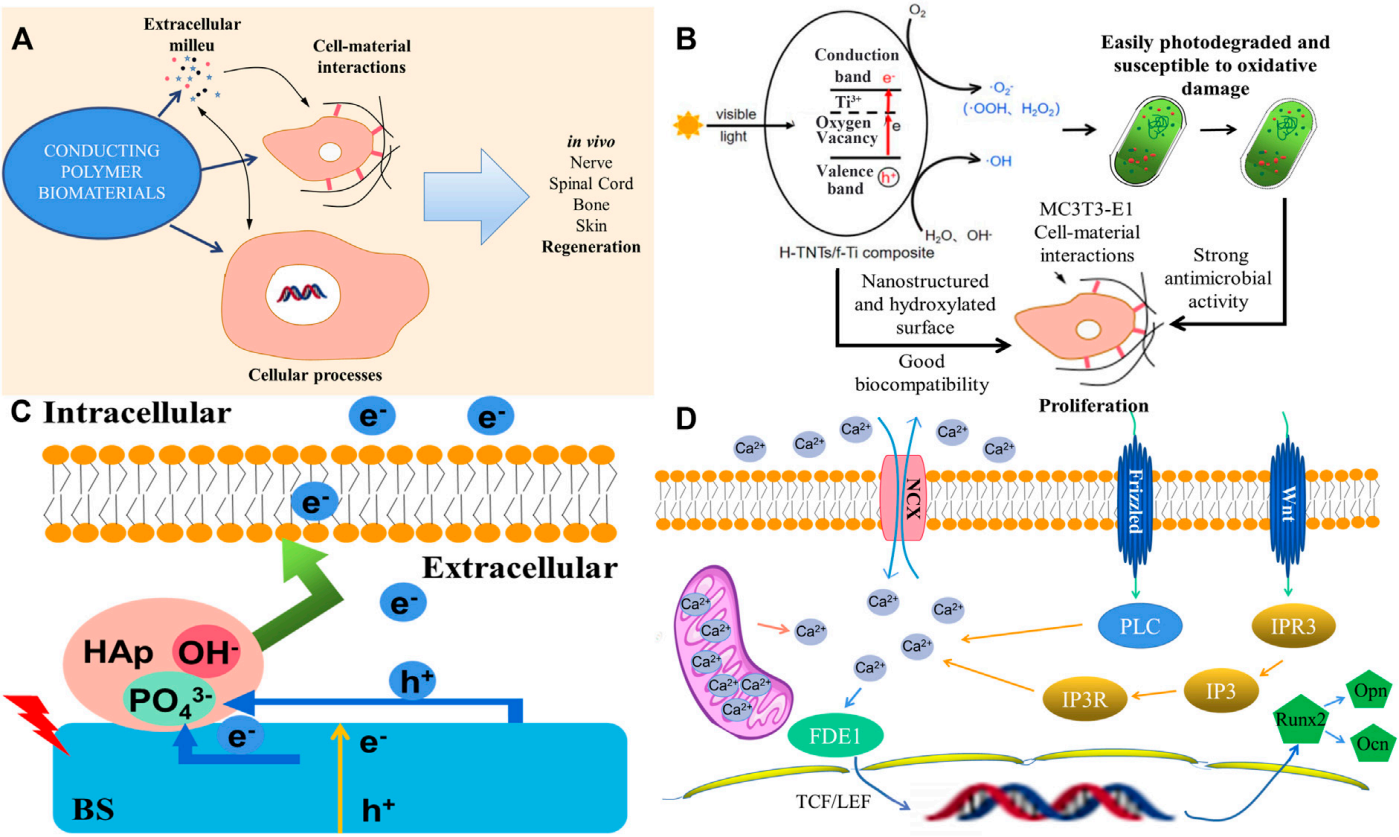
The mechanism and effect of photoelectric-responsive materials. **(A)** Conducting polymers and their effects on the tissue environment, cell outcomes, and *in vivo* regeneration. **(B)** Diagram of the interaction between photoelectrons and cells. **(C)** Diagram of the Ti-BS/HAp osteogenic differentiation mechanism under 808 nm irradiation. **(D)** Schematic representations of antibacterial activity for the H-TNTs/f-Ti composite under visible light irradiation (Fu et al., 2019; Petty et al., 2020; Zhao et al., 2021).

Optoelectronic materials are highly sensitive to NIR light, which can widely absorb and convert laser energy into electrical stimulation to activate the downstream signaling pathway. Photosensitive optoelectronic materials with good biocompatibility, such as polymers, bismuth sulfide (BS), manganese dioxide nanoparticles, and other materials with photothermal conversion efficiency, could achieve remote, precise, and noninvasive NIR irradiation and control cell differentiation behavior *in vitro* as well as tune the photoelectric microenvironment *in vivo* (Fu et al., 2019; Wang et al., 2020). Conducting polymers (CPs) have unique electroactive and photoelectric properties, such as bulk mixed electronic/ionic conduction, making it possible to manufacture multifunctional biomaterials those passively affect cell response, regulate the electric field, charge injection or drug delivery, and actively affect the process of tissue regeneration (Figure 4A) (Petty et al., 2020). A photoelectric-responsive material–hydrogenated TiO_2_ nanotube/Ti foil (H-TNT/f-Ti) composite with a higher visible photoelectric response and more hydroxyl functional groups has also been fabricated, which inhibits proliferation of *Streptococcus mutans* and *Porphyromonas gingivalis* which are the main cause of implant failures. Moreover, the proliferation of MC3T3-E1 cells on the hydroxylated surface was promoted, and improved biocompatibility with osteogenic cells was observed. As shown in Figure 4B, the photocurrent produced by the photoelectric response guarantees antibacterial activity and better biocompatibility with MC3T3-E1 cells for proliferation, thus providing a simple and effective method to significantly improve dental implant efficacy (Zhao et al., 2021).

A study used bismuth sulfide/hydroxyapatite (BS/HAP) film to create a rapid and repeatable photoelectric response microenvironment around the implant. Under NIR light irradiation, the corresponding increase in photocurrent on BS/HAP films was mainly attributed to the depletion of holes through PO_4_^3−^ from HAp and interfacial charge transfer by HAp compared with BS. Electrons activated the Na^+^ channels of mesenchymal stem cells (MSCs) and changed cell adhesion in the intermediate environment. RNA sequencing showed that when photoelectrons were transferred to the cell membrane, sodium flux and membrane potential depolarized and changed the cell shape. At the same time, calcium flux and FDE1 expression were upregulated. Moreover, TCF/LEF in the nucleus began to be transcribed and regulated the downstream genes involved in osteogenic differentiation through the Wnt/Ca^2+^ signaling pathway. In this study, NIR light–activated photoelectrons with BS/HAP film were transferred to the intracellular space controlled cell fate, directed osteogenic differentiation *in vitro*, and promoted bone regeneration *in vivo* (Figure 4C). The specific reaction mechanism is shown in Figure 4D. The Wnt/Ca^2+^ signaling pathway, a known membrane sensor and osteogenic marker, was upregulated in Ti-BS/HAp after light irradiation. Calcium then entered the cell nucleus to influence TCF/LEF through FDE1. Downstream genes related to osteogenic differentiation were activated. The Wnt/Ca^2+^ signaling pathway affects the intracellular Ca^2+^ concentration to modify osteogenic differentiation (Fu et al., 2019). However, although light is a relatively harmless energy source, it is mostly used for the regeneration and reconstruction of superficial tissues. Considering the limitation of penetration, frequent *in vitro* irradiation, and the complexity of biological tissue defects, the development of optoelectronic materials in the field of bone regeneration is limited to a certain extent.

### 3.3 Magnetoelectric materials

To overcome the penetrating limitation of optoelectronic materials, researchers switched their attention to magnetic fields, which have been put into clinical application and exhibit ideal penetration ability as well as minimal cytotoxicity on living tissues (Roessler et al., 2005).

The relationship between magnetism and polarization is the classical research content of physics. It is well known that the key physical property of magnetoelectric materials, or multiferroic materials, is the coupling between magnetism and polarization, that is, magnetoelectricity (Dong et al., 2019a). In solids, magnetism and electricity are derived from spin and charge degrees of freedom, respectively. In recent years, the intersection between these two fascinating topics has developed and become a new branch of condensed matter physics called magnetoelectricity (Fiebig et al., 2016; Dong et al., 2019b). The first phenomenon of the magnetoelectric effect was observed in the dielectric material, which was magnetized when passing through an electric field. Chun et al. (2010) reported that there was a large magnetoelectric (ME) effect in aluminum-substituted y-hexagonal Ba_0.5_Sr_1.5_Zn_2_(Fe_1−x_Al_x_)_12_O_22_. The amazing enhancement of ME effect by Al substitution could be preliminarily attributed to the reduction of magnetocrystalline anisotropy, which magnetizes along the plane perpendicular to the hexagonal axis. For the unique magnetoelectric effect, magnetoelectric materials can realize mutual transformation of the magnetic field and electric field and control electric polarization through a magnetic field or magnetic polarization through an electric field, which is widely used in magnetoelectric sensors, microwave devices, and magnetic recording.

Recently, an MNC-(AuNP RGD) heterodimer nanoswitch was developed, which was composed of AuNPs flexibly coupled to an RGD coating by a magnetic nanocage (Kang et al., 2018). This study provides initial evidence of physical and reversible ligand thawing for controlling stem cell adhesion through magnetic nanoswitches. Magnetic nanoswitches can also help regulate various cell functions *in vivo*. Moreover, such a physical, noninvasive, noncontact, and reversible nanoswitch can potentially improve the performance of material implants and promote the regenerative treatment of stem cells, such as osteogenic differentiation.

Magnetic nanoparticle techniques offer a number of important advantages over conventional, mechanically preconditioned, induced tissue engineering scaffolds, such as compression or fluid flow–based perfusion, by the precise control and duration of the levels of forces that can be applied to cells within a three-dimensional construct (Holtorf et al., 2005; Jafari et al., 2019). A new magnetic particle technology that allows highly local mechanical forces to be directly applied to a specific region of the ion channel structure was also reported. The results showed that manipulating the particles in the extracellular ring region extended for TREK-1 lead to a change in the whole cell current, which is consistent with the change in TREK-1 activity. follow-upKnowing the potential role of magnetic particle, follow-up studies should be carried out focusing on the usage as a tool to treat ion channel dysfunction caused by human diseases (Hughes et al., 2008). Furthermore, a study investigated remote magnetic field activation of magnetic nanoparticle–tagged mechanosensitive TREK-1 receptors on the cell membrane of human bone marrow stromal cells for use in osteoprogenitor cell delivery systems and the activation of *in vitro* and *in vivo* differentiation toward an osteochondral lineage, from which these cell manipulation strategies offer tremendous therapeutic opportunities in soft and hard tissue regeneration (Kanczler et al., 2010).

The aforementioned studies and reports do not clearly answer how the new generation of biomagnetoelectric materials control polarization through the magnetic field, from which the direct evidence of this association needs further exploration. However, the magnetic field, as a kind of mechanical stimulation, leads to the potential regulation of cells and triggers subsequent changes in whole-cell currents to promote osteogenic differentiation. In the future, the use of a magnetic field as the direct source of bioelectric stimulation acting on osteoblast-related cells is the direction we strive to explore.

### 3.4 Acoustoelectric materials

After considering physical, noninvasive, and noncontact external energy that can be converted into electrical energy, such as light energy and magnetic fields, researchers turn their attention to the strategy of sound-generated electricity. Compared with the external energy sources mentioned before, the tissue penetration ability and stability of ultrasonic waves are more desirable. Furthermore, as a kind of green energy, ultrasonic waves have less possible tissue toxicity and more availability because of economic effects.

Two-dimensional materials, such as graphene and molybdenum disulfide, are suitable for surface acoustic wave (SAW) device integration (Mas-Ballesté et al., 2011). The electric field associated with the propagation of the back wave on the piezoelectric substrate can be used to transmit carriers over a macro distance at the speed of sound in these materials, which results in an acoustoelectric (AE) current, a phenomenon in which electromotive force is produced by the action of sound waves propagating in semiconductors. This effect has been studied in other nanostructures which are applied to metrology and quantum information (Morocha and Rozhkov, 2018; Poole and Nash, 2018).

**FIGURE 5:**
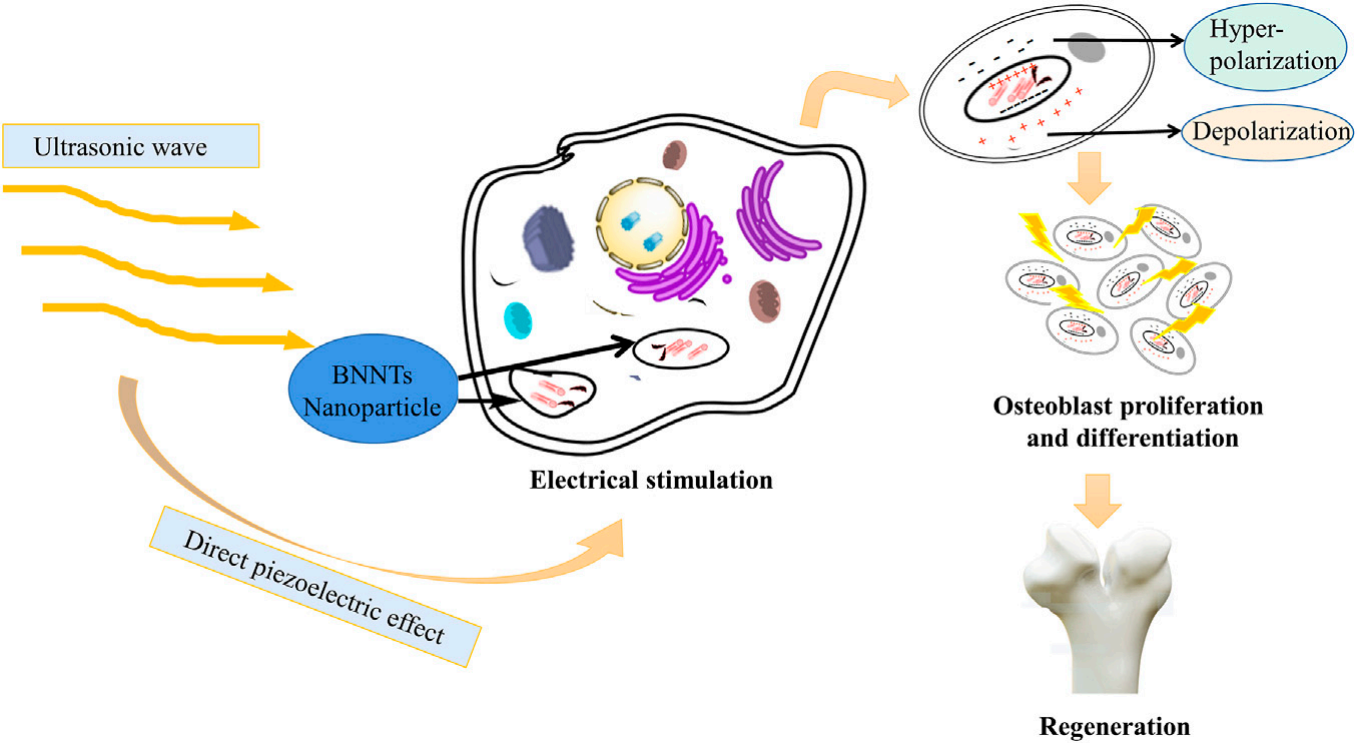
BNNT nanoparticles can internalize external ultrasonic irradiation into electrical stimulation. Under the direct piezoelectric effect, ultrasound, as a mechanical stress, is transformed into electrical stimulation and then promotes proliferation and differentiation of osteoblasts (Jacob et al., 2018).

To our knowledge, there is no specific literature to support that acoustoelectric materials have entered the field of tissue engineering at present. However, a few studies have proven the relationship between acoustoelectric and piezoelectric mechanisms. Herein, these basic theories and concepts of piezoelectric materials need to be further reviewed to understand the relationships between them. Under the applied mechanical pressure, the electrical characteristics of piezoelectric materials change with the deformation of crystal structure and shape. This phenomenon is called the positive piezoelectric effect. By contrast, when a voltage is applied to the crystal, its structure and shape will also change with it. This phenomenon is called the reverse piezoelectric effect. A number of acoustic and electroacoustic devices, such as crystal pickups, crystal oscillators, and loudspeakers, can be fabricated by utilizing the piezoelectric effect. Previous studies have indicated that when the longitudinal wave propagates in the semiconductor, additional periodic potential field waves are generated, and the period of the wave is the same as that of the sound wave. In atomic semiconductors, sound waves will produce a distorted periodic potential field, and the amplitude is small at this time. In a piezoelectric semiconductor, a sound wave will produce a piezoelectric periodic potential field, while the wave amplitude is very large. If an electron passes through at this moment, when the mean free path of the electron is smaller than the wavelength of the sound wave, the electron will continue to suffer from the scattering of phonons and lose energy such that the electron is captured by the trough of the periodic potential field generated by the sound wave. Meanwhile, when the sound wave propagates, the electron is pulled forward by the sound wave potential field and results in an electromotive force, which is the effect of “sound waves producing electrical effect” or acoustoelectric effect. This is the basic principle of applying the sound electricity effect to sound power generation (Morocha and Rozhkov, 2018; Ghosh, 2019; Mansoorzare and Abdolvand, 2019). Researchers use boron nitride (BNNT) as a nanocarrier to carry charged/mechanical stimulation on demand in the cell system. After the internalization of BNNTs, electrical stimulation is transmitted to tissues or cells through a wireless mechanical source (i.e., ultrasound). That is, the electric stimulation of cells is generated by internalized BNNT nanoparticles after external ultrasonic irradiation, which utilizes ultrasound as the mechanical stress on piezoelectric BNNTs to produce electrical stimulation to enhance cell differentiation (Figure 5) (Ciofani et al., 2008).

Because of the maturity of the theory of piezoelectric effect osteogenesis, the application of the acoustoelectric effect can creatively apply sound waves to generate bioelectricity to promote osteogenesis, in which the piezoelectric effect plays a bridge and intermediary role from “acoustic wave generates electricity” to “bone regeneration.” In brief, it is believed that acoustoelectric materials can be expected in the future.

### 3.5 Thermoelectric materials

Since the discovery of the Seebeck effect and Peltier effect in the early 19th century, the theoretical basis for the application of thermoelectric energy converters and thermoelectric refrigeration has been gradually enriched (Yamamoto et al., 2021). In the past few decades, thermoelectric materials (TEs) have attracted considerable attention in the consumer market due to their outstanding reliable, lightweight, and noiseless features (Lei et al., 2021; Zhou et al., 2021).

As the name suggests, TE can realize the mutual transformation between heat and electric energy. It is reasonable to consider whether heat energy could be converted, especially biological body temperature and external room temperature difference, into electric energy and further use energy that has been ignored in the field of tissue engineering. In terms of the implementation path, there is an obvious temperature gradient from the body nucleus to the body surface, which is the basis for the conversion of heat energy into electrical energy, and the generated microcurrent, which is a continuous and stable output under physiological adaptation, which further activates the proliferation and differentiation of osteoblasts through the above electroresponsive stimulation osteogenesis mechanism due to external stimuli-responsive bone therapy. It is an established fact that the classical theory of electrical stimulation osteogenesis has been demonstrated to accelerate bone regeneration and maintain BMSC stemness both in animal experiments and clinical practice by upregulating bone morphogenetic proteins under electrical stimulation, thus ultimately stimulating the calcium–calmodulin pathway, transforming growth factor-β (TGF-β), and other cytokines (Wei et al., 2022). In terms of the implementation scheme, the appropriate TE carrier should be the medium to accelerate osteogenesis. This requires the material itself to make full use of the temperature gradient of the human body or the temperature difference between the human body and its environment, to have a reasonable conversion efficiency that reaches the current threshold required for osteogenesis, to form a good closed circuit and overcome the extremely high thermal resistance of the environment between the human body and ambient air. To realize these requirements, researchers need to further understand and explore appropriate thermoelectric carriers (Kishore et al., 2019). Hence, the evaluation criteria and classification of thermoelectric materials will be briefly introduced in this review.

The thermoelectric performance of a material can be defined as the thermoelectric figure of merit ZT (ZT = S^2^σ T/κ) to evaluate, where “S” is the thermoelectric power or Seebeck coefficient, “T” is the absolute temperature, “σ” is conductivity, and “κ” is the thermal conductivity. To have a high thermoelectric merit ZT, the material must have a high Seebeck coefficient (S), electron conductivity, and low thermal conductivity (Xiao et al., 2014).

Electrothermal materials can be divided into three categories according to their operating temperature. First, bismuth telluride and its alloy are widely used in thermoelectric coolers with optimal operating temperatures less than 450°C. The second is lead telluride and its alloy, which are widely used in thermoelectric generators with an optimal operating temperature of approximately 1,000°C. The third is silicon germanium alloy, which is also widely used in thermoelectric generators with an optimal operating temperature of approximately 1,300°C (Chen et al., 2018; Feng et al., 2018).

**FIGURE 6:**
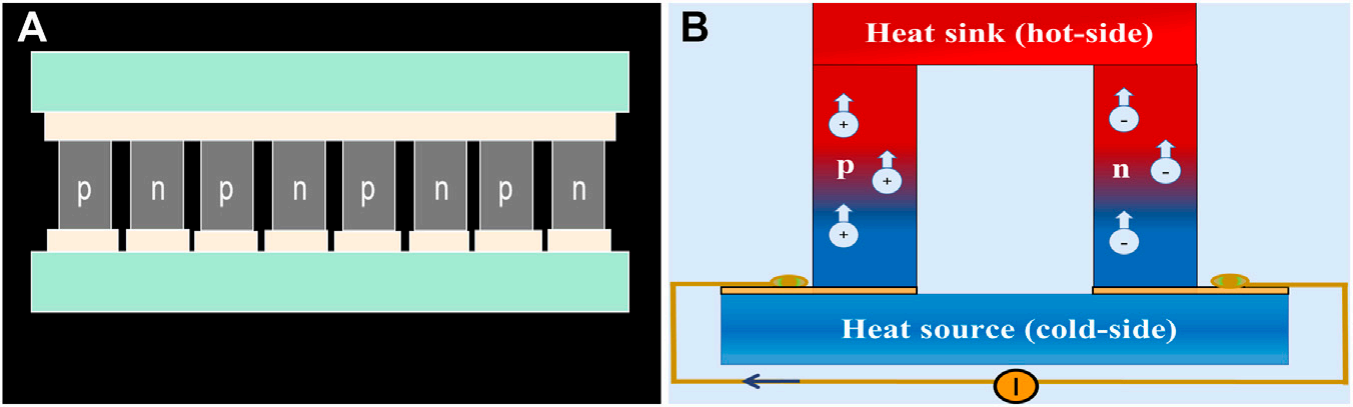
The construction and schematic diagram of TE materials. **(A)** TE materials consist of several thermocouples composed of p-type and n-type thermoelectric legs, which are electrically in series and thermally in parallel. **(B)** Once electric current is applied, charge carriers, electrons in n-type, and holes in p-type would transfer heat in the module from one side to the other (Kishore et al., 2019).

Due to the biocompatibility issue of lead-containing materials, restriction of the optimal operating temperature, cost performance of mass production, toxic organic solvents in the production process, and the absence of obvious temperature differences in the human body under constant temperature conditions, the clinical application of thermoelectric materials is limited to effectively converting heat energy into electric energy. However, some thermoelectric materials can generate microcurrents in a temperature environment. For example, highly robust and flexible thermoelectric (TE) films based on n-type Ag_2_Te nanoshuttle/polyvinylidene fluoride by solution treatment without surfactant are prepared, which achieves a favorable power performance of more than 30 μW (mK^2^)^−1^ at room temperature. In addition, the synthetic fabric also shows application potential in flexible electronic devices, as the performance change after 1,000 bending cycles can be ignored (Zhou et al., 2018). Unfortunately, TE is still infrequent in the field of bone defect regeneration, partly because the requirements for materials and conditions are rigorous, and both p- and n-type TE materials with equivalent performance are required to construct high-performance TE devices to form a closed loop, indicating that a p-type flexible TE counterpart is highly desirable (Figure 6A) (Snyder and Toberer, 2012).

At present, thermoelectric materials have already entered the field of medicine and health care. Embedding a thermoelectric generator (TEG) in a biological body is a promising way to supply electronic power in the long term for an implantable medical device (IMD). Through theoretical analysis, it has been found that the highest temperature gradient occurs near the skin surface of the human body, which suggests a candidate site for implanting and positioning the TEG. As the calculation shows, cooling the skin surface to 277 K can make the highest temperature difference reach up to 1.4 K. While heating the skin surface to 313 K, the maximum temperature difference is no more than 0.119 K (Yang et al., 2007).

In addition, using the temperature difference between the natural environment and the body surface is also a reasonable way to increase the temperature difference. The basic strategy of wearable TEGs is that after the current is applied, the carriers move while the charge carriers, holes in p-type, and electrons in n-type legs transfer heat from one side of the module to the other (Figure 6B). However, the skin temperature of the human body is different in different parts of the body and increases with increasing ambient temperature. During generations of natural evolution, human skin becomes a poor conductor of heat, as does ambient air, making TEGs operate in an extremely high thermally resistive environment; moreover, the shallow location may limit their scope of application (Kishore et al., 2019).

The future tendency is to synergistically optimize and integrate all the effective factors to further improve the TE performance such that highly efficient TE materials and devices can be more beneficial to daily lives (Yang et al., 2018).

## 4 Future prospective of electrogenesis bio-reactive materials

Piezoelectric and optoelectronic materials are the two earliest researched kinds of materials used in tissue engineering. Piezoelectric materials are widely used in the repair of load-bearing tissues. Although photoelectric materials are limited by phototoxicity, weak penetration ability, frequent irradiation by operators, and inappropriateness for deep wound repair, optoelectronic materials are more likely to be used for accurate stimulation of superficial tissues that are easily penetrated by near-infrared light. At present, except for piezoelectric and optoelectronic materials, a large number of experiments *in vivo* and *in vitro* have not proven to effectively promote bone tissue regeneration, which only stays in the theoretical stage, leaving a gap that we need to make an effort to fill in the future.

Piezoelectric materials are more mature in the field of electromechanical sensors than in tissue engineering. Nevertheless, it can be deduced that electroactive scaffolds can generate an electric field according to small mechanical vibrations and regulate the effective electric field characteristics of natural ECM observed during tissue development, regeneration, or repair. Photoelectric materials are only partially used for superficial tissue repair at present. Acoustoelectric and magnetoelectric materials, two materials with better penetration ability than photoelectric materials, also lack much clinical scientific research support. The thermoelectric material itself does not need an external invasive power supply and does not need frequent operation (such as irradiation) by the operator. Its mechanism is to use the temperature difference between the inherent body temperature and the external environment to further stimulate bone tissue repair. However, considering that the organism is a constant temperature condition, it is only suitable for the repair of defects under the condition of a possible temperature difference between the superficial and the body.

Indisputably, in addition to the necessary biocompatibility, the application of a new material should also consider biodegradability, physical and chemical stability, and properties that can simulate the microenvironment *in vivo* and be used as a platform for carrying “seed” cells and growth factors.

The osteogenesis potential of bio-reactive electrogenic materials encouraged the personalized and comprehensive synergetic application to make the utmost of their advantages and avoid their disadvantages. For instance, the combination of acoustoelectric and piezoelectric materials can comprehensively improve the bone repair efficiency than using one of them alone. Meanwhile, developing the process of preparing nanosized materials according to their characteristics and nanobioelectric active materials themselves will be more conducive to simulating the physiological microenvironment and promoting repair. Apart from the treatment of bone defects, electrostimulation can further modulate a myriad of biological processes, from cell cycle, migration, proliferation, and differentiation to neural conduction, muscle contraction, embryogenesis, and tissue regeneration. Recent advances in electroactive biomaterials are systematically overviewed for modulation of stem cell fate and tissue regeneration, which mainly include nerve regeneration, bone tissue engineering, and cardiac tissue engineering (Snyder and Toberer, 2012). When confronting the problems of premature degradation and insufficient performance of drugs and biomaterials, it is essential to build a suitable material carrier system for packaging and modification. Commercial products, such as alginates, gelatin, and chitosan, are widely used in biomedical applications such as drug delivery, cell encapsulation, anti-adhesion materials, and tissue engineering scaffolds because of their biocompatibility, providing a novel strategy to develop a suitable carrier to promote bone restoration.

As a representative of new advanced bone repair materials, bio-reactive electrogenic materials have the properties of being noninvasive, independent of external power supply, ergonomic, and as the product of the progress of materials science and physiology, showing favorable effects and bright prospects in tissue reparation and reconstitution (Table 1).

**TABLE 1:** Representative bio-reactive electrogenic materials and their electric constants.

Type	Representative material	Electric constant	Reference
Piezoelectric materials	PVDF	d_ij_ constant, 20 pC/N	Li et al. (2019a)
	HA/PVDF	d_ij_ constant, 1.5 pC/N	Wu et al. (2020)
	P(VDF-TrFE)	d_ij_ constant, 30 pC/N	Fukada (1998)
	Barium titanate	d_ij_ constant, 191 pC/N	Mindlin (1972)
	Potassium sodium niobate or lithium-doped potassium sodium niobate	d_ij_ constant	Yu et al. (2012)
		63 pC/N or 98 pC/N	
Optoelectronic materials	Bismuth sulfide/hydroxyapatite film	Photocurrent density, 25 μA cm^−2^ (under NIR light, 0.29 W cm^−2^)	Fu et al. (2019)
	Hydrogenated TiO_2_ nanotube/Ti foil	Photocurrent density, 4 μA cm^−2^ (under visible light, 100 mW cm^−2^)	Zhao et al. (2021)
Magnetoelectric materials	MNC–(AuNP RGD) heterodimer nanoswitch	Unstated	Kang et al. (2018)
Acoustoelectric materials	Graphene nanoribbons	Exhibiting linear dependence on surface acoustic wave intensity and frequency	Poole and Nash (2018)
Thermoelectric materials	Ag_2_Te nanoshuttle/polyvinylidene fluoride	Thermoelectric efficiency, 30 μW (mK^2^)^−1^	Zhou et al. (2018)

However, limited by the degradation rate, Young’s modulus, stiffness, efficiency, cytotoxicity to the body, the lack of appropriate carrier, the lack of economic effect, and the aftereffect observation of the materials, there are still inherent defects in the program of material processing and the materials themselves. Energy conversion materials such as thermoelectric and optoelectric materials are important to green energy systems, but at present, their energy conversion efficiency is relatively low. Ground-breaking design and fabrication technology for novel bioelectric active osteogenic materials will play an important role in the diagnosis and treatment of bone tissue defects.

## 5 Summary

In terms of interaction activity, the above bio-reactive electrogenic materials are utilized as carriers to convert physical stimuli such as mechanical pressure, sound waves, light waves, magnetic fields, and temperature differences into microcurrents and act on osteoblast-related cells through the established classical theory of electrical stimulation osteogenesis by upregulating bone morphogenetic proteins under electrical stimulation, thus ultimately stimulating the calcium–calmodulin pathway, TGF-β, and other cytokines. Although the corresponding biological evidence needs to be accumulated over time, the valuable role of bio-reactive electrogenic materials would be self-evident. Overall, an appropriate material carrier is vital for the output current for osteogenesis under physiological conditions, which requires a large number of experimental explorations and subsequent staged clinical trials in the future.
